# Knowledge, Attitudes and Perceptions of Saudis towards Participating in Clinical Trials

**DOI:** 10.1371/journal.pone.0143893

**Published:** 2016-02-05

**Authors:** Mohamad A. AL-Tannir, Nahid El-Bakri, Amani K. Abu- Shaheen

**Affiliations:** Research Center, King Fahad Medical City, Riyadh, Saudi Arabia; The George Washington University School of Medicine and Health Sciences, UNITED STATES

## Abstract

**Aim:**

To assess the knowledge, attitudes, and perceptions of Saudis towards participating in clinical trials (CTs).

**Methods:**

A cross-sectional study was conducted on 232 Saudi adult patients and their companions visiting adult outpatient clinics at King Fahad Medical City, Riyadh, Saudi Arabia. Data were collected using a self-administered questionnaire based on information obtained from the literature. The questionnaire was divided into four sections, one covering the respondents’ demographics, and the other three assessing knowledge, attitudes, and perceptions towards participating in CTs.

**Results:**

A total of 148 (63.8%) respondents were males, and 52 (22.4%) participants had been invited to participate in a CT previously. Of those, 39 (75%) participated. Knowledge about the essential elements of informed consent ranged from 55.7% (number of participants needed) to 85.7% (confidentiality of personal information). The majority (163, 73.8%) of respondents was willing to participate in a CT after consulting their family physician and 130 (58.0%) respondents would be motivated to participate in a CT if they were healthy. Only 36.8% of the respondents believed that patients who participated in a CT received the best care. Moreover, 110 (48.7%) respondents believed that research was conducted in a responsible and ethical manner.

**Conclusions:**

The present study assessed the current understanding of CTs among Saudi participants. Although the majority of participants had an acceptable level of knowledge about CTs, they exhibited conditional attitudes and misperceptions towards participating in a CT. Increased patient awareness may improve patients’ attitudes towards ethical conduct of CTs.

## Introduction

Clinical trials (CTs) are essential for identifying effective therapies in modern medicine [1]. When properly conducted, CTs are the fastest and safest way to determine which therapeutic strategies and diagnostic tests are most effective. As CTs require human subjects, ethical approval is mandatory to ensure safe and successful execution of a clinical trial (CT). Despite the high level of safety being given to attract participants for CTs, recruitment has always been a challenge, and most people remain unaware of CTs and how their participation contributes to the development of future drugs and devices [2].

Some factors that affect willingness to participate in a CT include anticipated benefits from participating, patient understanding of what is required from them, and the level of trust patients place in investigators [3,4]. In addition, the majority of participants in CTs are reluctant to do additional monitoring tests, particularly those that are invasive, as they can be associated with potential morbidity or may be inconvenient for the patient [5]. Previous studies have identified factors that affect enrollment in oncology trials, such as geography, a desire for non-investigational therapies, fear of randomization, age, socioeconomic status, and educational level [5]. In contrast, patients who have prior experience with participating in a CT may be easily motivated to participate again. Knowledge of risks, transparency of information, and addressing concerns of eligible participants enhance their overall trust with the investigators [4]. In fact, studies on physician-patient communication have reported that patients who feel that the study objectives have been clearly communicated to them are more likely to participate [6–10].

Several studies have been performed to assess the perceptions of patients regarding CTs from the US, Denmark, Australia, and Japan [11–14]. However, these results may not be applicable to other countries with different sociocultural backgrounds; consequently it is important to consider social, cultural, and economic perspectives when a study is designed [15,16]. Currently, there is limited empirical research involving the knowledge, attitudes, and perceptions of individuals from developing countries and the Middle East about CTs [17, 18]. Accordingly, further studies would be useful to clarify interests and concerns regarding participation of individuals from these countries in CTs. CTs are not as common in Saudi Arabia, compared to developed nations. Therefore, the current study was conducted to assess the knowledge, attitudes, and perceptions of Saudis towards participating in a CT.

## Materials and Methods

### Study design

This cross-sectional study was conducted at King Fahad Medical City (KFMC), a tertiary hospital in Riyadh in central region of Saudi Arabia in 2013. Ethical approval was obtained from the Institutional Review Board at KFMC. Participants who met the inclusion criteria were asked to participate in this study; those who agreed to take part gave written informed consent.

### Study population

Participants were adult Saudi patients and their companions visiting outpatient clinics at KFMC who could read Arabic and were willing to participate in the study. Exclusion criteria included age < 18 years old or visiting a psychiatry clinic.

### Recruitment

We approached and invited 300 adult Saudi patients and their companions from the waiting rooms of the outpatient clinics at KFMC over a one-week period to reach our sample size requirement of 232 participants. Of these, 68 declined to participate due to time limits. Adults were approached by a trained research coordinator to sign informed consent and to complete a questionnaire.

### Data collection

Data were collected using a self-administered questionnaire based on information obtained from the literature to assess the knowledge, attitudes, and perceptions towards participating in a CT [12, 18, 19]. The questionnaire was developed in the Arabic language, and a pilot study was conducted with 30 participants to estimate knowledge about CTs among Saudi adults. The questionnaire was revised according to their comments. The questionnaire was divided into four sections. Section 1 included age, gender, educational level, marital status, and history of any chronic disease. Section 2 explored the respondents’ knowledge about the potential benefits of a CT, essential elements of informed consent and what a CT includes. Section 3 assessed participants’ attitudes concerning willingness to participate in a CT, and section 4 explored respondents’ perceptions towards participating in a CT. A 5-point Likert scale was used for the perceptions' and attitudes' questions (“strongly agree”, “agree”, “uncertain”, “disagree”, and “strongly disagree”).

### Sample size estimate

A pilot study of 30 participants was conducted to estimate knowledge about CTs among Saudi adults. The results showed that 22 (82%) participants had some knowledge regarding CTs compared to 65–75% of participants in European and North American countries. This result enabled us to calculate the required sample size of 232 participants. Sample size was calculated using the Raosoft online sample size calculator with a 95% confidence interval and a 5% margin of error.

### Statistical analysis

Data analysis was conducted using SPSS 20.0 software (SPSS Inc., Chicago, IL, USA). Continuous variable are presented as means with corresponding standard deviations or as medians with corresponding ranges, as appropriate. Categorical variables are presented as frequencies with corresponding percentages. We used descriptive and chi-square analyses to determine the strength of the association between independent variables (age, gender, educational level, marital status, and chronic disease) and the main outcome variable of interest. To enhance our analyses, we collapsed education level into subgroups of high school or less, university, and postgraduate. To improve power, we collapsed Knowledge question responses into “yes” and the combination of “no” and “I don’t know”. The Likert scale responses were collapsed into combinations of (1) “strongly agree” and “agree” and (2) “uncertain”, “disagree”, and “strongly disagree”.

## Results

Respondents’ demographics are presented in Table 1. A total of 148 (63.8%) respondents were males. The age of responders was 18–70 years (mean, 33.24 ±10.76 years). More than half (55.6%) of the respondents had achieved university level of education. Fifty-two (22.4%) participants have been invited to participate in a CT previously, and 39 (75%) had agreed to participate.

**Table 1:** Independent variables of respondents.

Demographic variables	
**Mean age years**	33.24 (±1.1)
**Median age years**	30.50 [18–70]
**Gender**	
**Male**	148 (63.8)
**Female**	84 (36.2)
**Education Level**	
**High-school-or-less**	86 (37.4)
**University**	129 (56.1)
**Postgraduate**	15 (6.5)
**Marital status**	
**Married**	157 (67.7)
**Single**	68 (29.3)
**Divorced**	7 (3.0)
**Having chronic disease**	
**Yes**	59 (25.4)
**No**	173 (74.6)

Data are presented as either mean (±SD), median [Min-Max] or actual numbers (%).

### Knowledge of respondents towards participating in a CT

Our results after asking participants about the potential benefits of CTs indicated that the majority (195, 85.9%) of respondents stated that CTs improve medical knowledge, whereas 19 (10.0%) respondents indicated that CTs have no benefit. Moreover, 21 (9.1%) respondents were unaware of CTs (Table 2).

**Table 2:** Knowledge of respondents about clinical trials.

	Response n (%)
**What clinical trials can include**	
**Filling survey**	33(14.3)
**Implementing new test**	28(12.1)
**Testing new drug**	15(6.5)
**Testing new device**	6(2.6)
**All of the above**	128(55.4)
**I don’t know**	21(9.1)
**Potential benefits of clinical trials**	
**Improve medical knowledge**	195 (85.9)
**Improve patient care**	189 (83.6)
**Improve community well-being**	112 (50.9)
**No benefit**	19 (10.0)

Data are presented as actual numbers (%).

Table 3 shows the extent of agreement of respondents with the rights embedded in the essential elements of informed consent. Knowledge about the essential elements of informed consent ranged from 55.7% (number of participants needed) to 85.7% (confidentiality of personal information). Moreover, 160 (72.4%) respondents recognized that participation is voluntary, whereas 131 (59.5%) were aware of their right to withdraw from a CT at any time without consequences.

**Table 3:** Knowledge of respondents towards essential elements of informed consent.

Statement	Response n (%)	
	Yes	No
**Confidentiality of personal information**	191 (85.7)	32 (14.3)
**Anticipated benefits**	181 (81.9)	40 (18.1)
**Foreseeable risks**	176 (80.0)	44 (20.0)
**Alternative procedures or courses of treatment**	169 (76.5)	52 (23.5)
**Research aim**	168 (75.0)	56 (25)
**Voluntary nature of participation**	160 (72.4)	61 (27.6)
**Possible compensation**	143 (66.2)	73 (33.8)
**Right to withdraw and its consequence**	131 (59.5)	89 (40.5)
**Number of participants needed**	122 (55.7)	97 (44.3)

Data are presented as actual numbers (%).

### Attitudes of the respondents towards participating in a CT

When asked about the most important elements that would enhance participation in a CT, 89.7% of participants responded that they might participate if they understood the study. Moreover, 155 (71.4%) participants would participate if they signed informed consent. Approximately 74% of respondents were willing to participate in a CT after consulting their family physician, and 130 (58.0%) respondents would be motivated to participate in a CT if they were healthy (Table 4).

**Table 4:** Attitudes of study population towards participating in clinical trials.

	Agreed n (%)
**Factors associated with willingness to participate in clinical trials**	
**Take more time to think before approving**	173 (78.3)
**Consultation of family physician**	163 (73.8)
**Researchers are willing to participate in the same study**	136 (61.3)
**Presence of family members**	92 (41.6)
**Elements enhancing participation in clinical trials**	
**Understand the study**	200 (89.7)
**Researcher had explained the study**	191 (86.4)
**Family physician read the protocol**	177 (81.6)
**Signing informed consent form**	155 (71.4)
**Clinical conditions motivating participation in clinical trials**	
**Healthy status**	130 (58.0)
**Life threatening disease**	101 (46.3)
**None life threatening disease**	77 (35.3)
**Feeling offended if asked to participate in clinical trials during a regular doctor visit**	35 (15.6)

Data are presented as actual numbers (%).

### Perceptions of respondents towards participating in a CT

When asked about the reasons for participating in a CT, the three top reasons were: to help society (205, 92.3%), to help advance medical knowledge (198, 89.6%), and to benefit others through their participation (190, 87.2%).

Only 36.8% of respondents believed that patients who participated in a CT received the best care. Moreover, 110 (48.7%) respondents believed that research was conducted in a responsible and ethical manner (Table 5).

**Table 5:** Perceptions of study population towards participating in clinical trials.

Statement	Agreed n (%)
**Reasons to participate in clinical trials**	
**Helping the society**	205 (92.3)
**Help in advancing the medical knowledge**	198 (89.6)
**Others may benefit from participation**	190 (87.2)
**Helping in developing new medications**	183 (83.9)
**Receiving best medical care**	176 (80.4)
**Getting financial compensation**	57 (26.5)
**Reasons not to participate in clinical trials**	
**Fear from risks of participation**	134 (60.6)
**Fear from the unknown**	129 (59.2)
**Medical reasons**	104 (48.4)
**Mistrust the medical system**	81 (37.5)
**No financial compensation**	61 (28.2)
**Moral reasons**	54 (25.0)
**Patients who participate in clinical trial get the best care**	84 (36.8)
**Participation in clinical trials could cause patient exhaustion**	72 (31.9)
**Opinion about clinical trials conductions**	
**Clinical trials are conducted in a responsible and ethical manner**	110 (48.7)
**I don’t have an opinion regarding clinical trials**	83 (36.7)
**Clinical trials are conducted by unqualified personnel**	23 (10.2)
**Clinical trials are conducted in unethical manner**	10 (4.4)

Data are presented as actual numbers (%).

### Comparison between participants who had previously participated in a CT and those who had never participated

All items in the questionnaire were tested to compare the knowledge, attitudes, and perceptions between respondents who previously participated in a CT and those who had never participated in a CT. Knowledge, attitudes, and perceptions towards participating in a CT were significantly higher among respondents who had previously participated in a CT than those who had not (Table 6).

**Table 6:** Comparison between participants who had previously participated in a CT and those who had never participated in terms of knowledge, attitudes and perceptions towards participating in clinical trials.

Statement	Previously participated in clinical trials		p-value
	Yes (N = 39) n (%)	No (N = 193) n (%)	
**Reasons not to participate in clinical trials**			
**Fear from risk of participation**	15 (40.5)	119 (64.7)	0.006
**Fear of the unknown**	17 (44.7)	112 (62.2)	0.046
**Awareness of legal protection of participants in clinical trials**	15 (39.5)	36 (19.7)	0.008
**Clinical conditions motivating participation in clinical trials**			
**Healthy status**	29(74.4)	101(54.6)	0.023
**None life threatening disease**	19(51.4)	58(32.0)	0.025

Data are presented as actual numbers (%).

## Discussion

Our results reveal various valuable insights regarding the knowledge, attitudes, and perceptions of Saudis toward CTs. The respondents showed a satisfactory level of knowledge about the benefits of CTs, what CTs include, and the essential elements of informed consent. They showed a conditional attitude to participate in a CT. Another important observation was that only 48.7% of the respondents perceived that CTs are conducted ethically. Our findings matched our hypothesis that we expected the knowledge to be of optimum level as there is much awareness efforts and improvement in education level that was evident in our demographic data. Moreover, the conditional attitude and perceptions towards participating in a CT reflected the lack of CTs conducted in our area. It has been found that the Middle East and North Africa, only < 1% of global CTs [20]. Our findings suggest that respondents’ knowledge is related to subjects' protection and the basic nature and importance of CTs. Nevertheless, their attitudes towards participation were conditional, reflecting a misperception.

In accordance with our results, a satisfactory level of knowledge about CTs was reported previously by Bergenmar *et al*. who investigated the level of knowledge and perceived understanding among patients who participated in cancer CTs [21]. However, in contrast to our results, one study showed limited knowledge about several CT concepts [22]. A possible explanation for this difference is that they addressed more detailed methodological questions considering bias.

The respondents in our study showed a conditional attitude towards participating in a CT. We also found that the theme of trust was an important consideration for individuals invited to participate in a CT. As physicians are often important sources of information for patients making decisions regarding participation in a CT, approximately 74% of the respondents mentioned that they would need to consult with a physician involved in their care before participating in a CT. Similarly, a study by Tanai *et al*, concluded that CT design and the doctor-patient relationship may have a decisive impact on patient participation in a CT [23]. Such findings clearly indicate that a relationship built on trust with healthcare providers is essential not only for medical care but also for future CTs. Moreover, most respondents felt confident to participate if they signed informed consent. However, patients might adopt a more passive role during the informed consent process and seek less information regarding the study, particularly if trust between the patient and physician is high.

Our study also showed that respondents would feel more confident to participate in a CT if they understood and read the study protocol and if the investigator adequately explained the study design. Thus, it is important that investigators consider these attitudes and try to enhance patients’ understanding of the specific research questions of the CT. Fallowfield *et al*. reported that providing additional information increases willingness to participate in a CT [24]. However, other studies have shown that research participants do not understand research concepts even when the research staff clarifies the concepts [9, 25, 26].

The majority of respondents considered helping society to be a reason to participate in a CT. Moreover, they had misperceptions about the quality of care, as only 36.8% of respondents believed that study participants receive the best possible care. Furthermore, only 48.7% believed that CTs are conducted ethically. Perceptions and beliefs are the major factors limiting recruitment for research [27, 28]. Casseileth reported that 52% of research participants stated that their main reason for participating was to receive the best medical care, whereas only 13% believed that research participants receive better treatment [28]. Therefore, investigators need to provide further assurances that all necessary procedures will be used to minimize risks and enhance the protection of patients’ rights and well-being.

An important strength of the present study was that our sample included participants who had previous experience with a CT either by accepting or by declining to participate in a CT. Consequently, our results are representative of different insights regarding the knowledge, attitudes, and perceptions of adult Saudis towards participating in a CT. Our results will serve as the basis for future research and contribute to developing and optimizing strategies to combat these problems, such as educating patients or the community about CTs. The only study limitation was that our results may not be generalizable to other Saudis with lower education levels. Indeed, our participants were mostly educated, married men; thus, their motivation to participate in a CT may differ from other populations with different demographic characteristics. Our results are likely representative of the knowledge, attitudes, and perceptions toward participating in a CT of adult Saudis inhabiting central region of Saudi Arabia.

## Conclusions

The present study assessed the current understanding of CTs among Saudi participants. Although the majority of participants had an acceptable level of knowledge about CTs, they exhibited conditional attitudes and misperceptions towards participating in a CT. Increased patient awareness may improve patients’ attitudes towards ethical conduct of CTs.
